# The yin and yang of leishmaniasis control

**DOI:** 10.1371/journal.pntd.0005529

**Published:** 2017-04-20

**Authors:** Shaden Kamhawi

**Affiliations:** Laboratory of Malaria and Vector Research, National Institutes of Health, Rockville, Maryland, United States of America; Yale School of Public Health, UNITED STATES

## Background

The relevance of neglected tropical diseases (NTDs) to global poverty and political instability has ignited interest in this group of diseases with the emergence of terms and concepts such as ‘One Health’ and ‘Leaving no one behind’ [1]. Importantly, this led to the materialization of concrete programs and initiatives aiming at control, elimination or even eradication of some NTDs, most notable being the World Health Organization (WHO) Roadmap to overcome the global burden of NTDs (www.who.int/neglected_diseases/NTD_roadmap_2012_fullversion.pdf) and the London Declaration on NTDs (http://unitingtocombatntds.org/).

Leishmaniasis is a vector-borne NTD transmitted by the bite of infected phlebotomine sand flies. It afflicts an estimated 900,000–1.3 million people annually and is responsible for up to 30,000 deaths each year (http://www.who.int/mediacentre/factsheets). At present, leishmaniasis is undergoing a tug of war between natural phenomena and man-made conditions that facilitate the spread of disease, and our efforts to control it through rapid advances in knowledge, technology and communication. Here, I put forward some of the major interrelated yet opposing forces that are likely to shape the future of leishmaniasis control.

## The yin: Challenges facing leishmaniasis control

**Fig 1:**
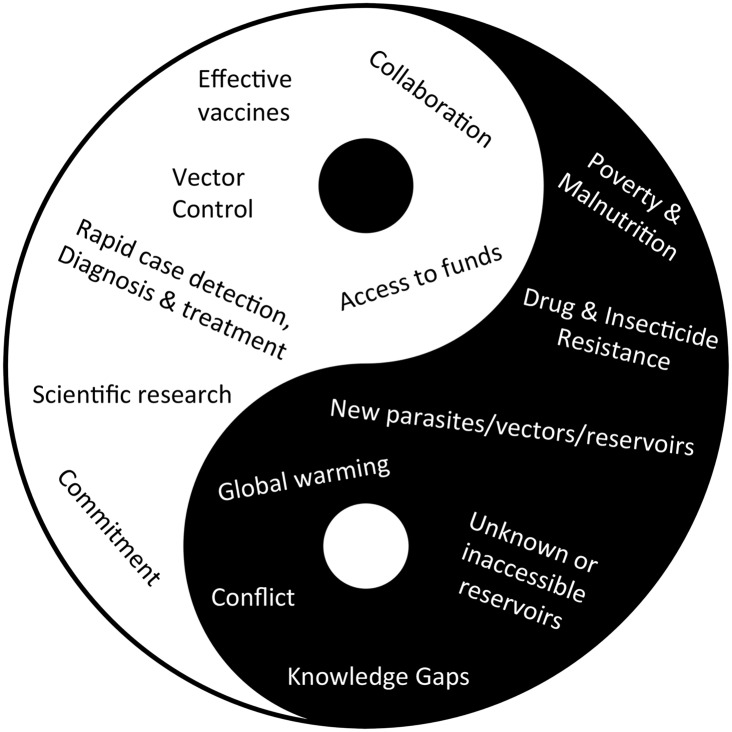
The yin and yang of leishmaniasis control. Disruptive natural and man-made disease-promoting elements are opposed by efforts to control or eliminate leishmaniasis through commitment, collaboration, and continued research towards better vaccines, drugs and diagnostics.

As a NTD, a zoonosis, and a vector-borne disease, leishmaniasis faces formidable obstacles to its control on a global scale (Fig 1). Each of these characteristics carries specific challenges making leishmaniasis one of the toughest diseases to control or eliminate. As a NTD, leishmaniasis is mostly an affliction of the poorest populations [1, 2] and is unlikely to have access to funding at the magnitude or stability reserved for research and development of diseases such as cancer, diabetes or HIV that afflict wealthy nations. As a zoonosis, leishmaniasis control is burdened by the presence of various animal reservoirs, some wild and inaccessible, forming a barrier to its elimination in the near future. As a vector-borne disease, fundamental aspects of both sand fly and human behaviors challenge vector control efforts [3], while increasing conflicts and global warming conspire to promote the persistence and spread of leishmaniasis. Outbreaks and epidemics of cutaneous and visceral leishmaniases have been reported as a consequence of massive population displacements when naïve populations become exposed to infected vectors, or when infected individuals encounter susceptible vectors. This is exemplified by current events in the Middle East and South Sudan [4, 5]. On the other hand, reports of autochthonous cases of leishmaniasis from countries such as Germany and Austria [6–8], and outbreaks in new foci in Latin America [9, 10], blamed in part on climate change, are slowly redrawing the global distribution map for leishmaniasis. In addition to the expansion of known forms of leishmaniasis, emergence of new disease manifestations, new vectors or new potential reservoirs have been recently reported [11–16] and may pose further challenges for control efforts.

## The yang: Progress towards leishmaniasis control

Though leishmaniasis control remains elusive to date, a fundamental change in the perception and awareness of power brokers and decision makers -*governments*, *non-profit organizations*, *for profit drug companies*, *and philanthropists*- have focused efforts on programs targeting the control or elimination of leishmaniasis in geographically contained regions (Fig 1). One of the most visible is a collaborative effort towards elimination of visceral leishmaniasis (VL) as a public health problem in the Indian sub-continent through a multidisciplinary and integrative approach ranging from case detection and treatment to vector control [3, 17, 18]. The WHO in partnership with member states (India, Bangladesh and Nepal), the Bill & Melinda Gates Foundation, Drugs for Neglected Diseases *initiative*, the KalaCORE Consortium among others have committed resources, expertize and funds to eliminate VL -*defined as a reduction in the annual incidence of VL to <1 per 10*,*000 cases per endemic block*- in the Indian subcontinent by 2020 [18]. Overall, this endeavor has been a success story with Nepal reaching and sustaining its target elimination, and Bangladesh and India reporting elimination status in 90% and >70% of endemic blocks, respectively [17, 18]. Yet, some scientists caution that VL epidemics in the Indian subcontinent have fluctuated cyclically prior to the start of the elimination program with cases peaking to epidemic proportions every 15 years before declining in response to multiple factors [19]. Therefore, care should be used to interpret the reduction observed in the number of cases since the inception of the elimination program. More important, this merits careful preparation for continued surveillance to insure that disease elimination is sustained long after the official conclusion of the program [20]. This will be the most challenging part of controlling or eliminating leishmaniasis anywhere; the ever present pressure of reemergence and the essentiality of continuous surveillance backed by a rapid response in regions where the disease is considered as ‘eliminated’ or ‘controlled’.

The choice of the Indian subcontinent by WHO and partners as an initial target for elimination of VL makes sense. VL is the most serious of leishmaniasis forms and the third deadliest parasitic disease [21], while its anthroponotic status in the Indian subcontinent, with humans as the only established disease reservoirs, makes it the most amenable for elimination. Yet, even with the assumption that the disease is a true anthroponosis -*unknown animal reservoirs may yet be revealed*- the gap in our knowledge of the most relevant source/s of infection for vector sand flies -*is it active VL cases*, *asymptomatic individuals or cured cases with post-kala azar dermal leishmaniasis*?- threatens the long-term success of elimination [3, 17]. As for zoonotic visceral and cutaneous leishmaniases, merely controlling the disease is a challenge, complicated by the ability of parasites to persist in animal reservoirs independent of human infection. In these conditions, the only feasible long-term potential for elimination of human disease is development of effective vaccines, a solution for all forms of the disease, be it zoonotic or anthroponotic.

## Future directions: Maximizing the success of leishmaniasis control

To insure that we stay ahead of factors favoring the expansion and emergence of leishmaniasis, it is clear there is a critical need for continued investment in basic research focused on addressing questions relevant for leishmaniasis control. A major focus should be on vaccine development. Unfortunately, effective vaccines against human leishmaniasis have been elusive and not as easily attainable as was anticipated by the long-life immunity conferred by certain healed *Leishmania* infections [21]. In reality, characteristics of cutaneous and visceral leishmaniases caused by different parasite species are distinct. A healed infection by *L*. *major* usually confers immunity while infection with *L*. *donovani* needs to be treated if clinically patent and recurrent infections are common. This led to exploration of the concept of cross-protection from a serious form of leishmaniasis through immunity to a more benign form. Indeed, in an animal model of infection protection from VL was achieved by generating a robust immunity to cutaneous leishmaniasis [22]. This promising approach relies on live vaccination to promote protection. Evidence accrued from leishmanization of humans and from animal studies also indicate that a robust protective immunity may only be achievable after healing from a live virulent challenge [21, 23]. This supports the rationale for live vaccines and suggests that a single antigen vaccine may not be sufficient to confer immunity against leishmaniasis. This is further underscored by a previously unappreciated complexity to the egested infectious inoculum of a sand fly. It is now established that several vector-derived virulence factors—*to date known vector-derived virulence factors include the promastigote secretory gel*, *exosomes and components of saliva*- contribute to initial survival and establishment of parasites following sand fly transmission of *Leishmania*, and are thus an integral part of disease pathogenesis [24–26]. Taking this into consideration, it is not implausible that an effective vaccine against leishmaniasis would also benefit from the inclusion of some of these components to overcome the enhanced virulence of vector-initiated leishmaniasis, a notion that has recently been embraced by the vaccinology community [21, 27]. In addition to prioritizing vaccine research, we need new drugs and insecticides to stay ahead of emerging resistance in both parasites and vectors alike while optimizing the current use of diagnostics and combination drug therapies [17].

The global elimination of leishmaniasis in the absence of effective vaccines is an unrealistic expectation. As such, support for research directed at finding effective vaccines needs to be sustained, if not augmented. In the mean time, the only means available to outpace natural and man-made conditions that are advancing the spread of leishmaniasis is engagement at all levels from government to community, and commitment to uninterrupted surveillance, safeguarded by a practical fiscal plan that guarantees funding for these long-term activities. Only nurturing self-sufficiency within endemic countries can fulfill this ambitious target.

**Key points**

- As a neglected vector-borne disease, control of leishmaniasis faces formidable challenges.
- Elements such as climate change, population instability and globalization are driving the expansion of leishmaniasis.
- Advances in scientific research combined with commitment by governments and non-profit organizations are striving to tip the balance towards leishmaniasis control.
- Engagement of governments and communities of endemic countries is vital for long-term success of control efforts.
- Development of effective vaccines remains the optimal solution for the elimination of leishmaniasis on a global scale.
